# Effect of 10-valent pneumococcal conjugate vaccine on the incidence of radiologically-confirmed pneumonia and clinically-defined pneumonia in Kenyan children: an interrupted time-series analysis

**DOI:** 10.1016/S2214-109X(18)30491-1

**Published:** 2019-02-14

**Authors:** Micah Silaba, Michael Ooko, Christian Bottomley, Joyce Sande, Rachel Benamore, Kate Park, James Ignas, Kathryn Maitland, Neema Mturi, Anne Makumi, Mark Otiende, Stanley Kagwanja, Sylvester Safari, Victor Ochola, Tahreni Bwanaali, Evasius Bauni, Fergus Gleeson, Maria Deloria Knoll, Ifedayo Adetifa, Kevin Marsh, Thomas N Williams, Tatu Kamau, Shahnaaz Sharif, Orin S Levine, Laura L Hammitt, J Anthony G Scott

**Affiliations:** a KEMRI-Wellcome Trust Research Programme, Kilifi, Kenya; b Department of Infectious Disease Epidemiology, London School of Hygiene & Tropical Medicine, London, UK; c Aga Khan University Hospital, Nairobi, Kenya; d Oxford University Hospitals NHS Foundation Trust, Oxford, UK; e Imperial College, London, UK; f Kilifi County Hospital, Kilifi, Kenya; g Oxford University, Oxford, UK; h Department of International Health, Johns Hopkins Bloomberg School of Public Health, Baltimore, USA; i INDEPTH Network, Accra, Ghana; j Ministry of Health, Nairobi, Kenya; k The Bill & Melinda Gates Foundation, Seattle, WA, USA

## Abstract

**Background:**

Pneumococcal conjugate vaccines (PCV) are highly protective against invasive pneumococcal disease caused by vaccine serotypes, but the burden of pneumococcal disease in low-income and middle-income countries is dominated by pneumonia, most of which is non-bacteraemic. We examined the effect of 10-valent PCV on the incidence of pneumonia in Kenya.

**Methods:**

We linked prospective hospital surveillance for clinically-defined WHO severe or very severe pneumonia at Kilifi County Hospital, Kenya, from 2002 to 2015, to population surveillance at Kilifi Health and Demographic Surveillance System, comprising 45 000 children younger than 5 years. Chest radiographs were read according to a WHO standard. A 10-valent pneumococcal non-typeable *Haemophilus influenzae* protein D conjugate vaccine (PCV10) was introduced in Kenya in January, 2011. In Kilifi, there was a three-dose catch-up campaign for infants (aged <1 year) and a two-dose catch-up campaign for children aged 1–4 years, between January and March, 2011. We estimated the effect of PCV10 on the incidence of clinically-defined and radiologically-confirmed pneumonia through interrupted time-series analysis, accounting for seasonal and temporal trends.

**Findings:**

Between May 1, 2002 and March 31, 2015, 44 771 children aged 2–143 months were admitted to Kilifi County Hospital. We excluded 810 admissions between January and March, 2011, and 182 admissions during nurses' strikes. In 2002–03, the incidence of admission with clinically-defined pneumonia was 2170 per 100 000 in children aged 2–59 months. By the end of the catch-up campaign in 2011, 4997 (61·1%) of 8181 children aged 2–11 months had received at least two doses of PCV10 and 23 298 (62·3%) of 37 416 children aged 12–59 months had received at least one dose. Across the 13 years of surveillance, the incidence of clinically-defined pneumonia declined by 0·5% per month, independent of vaccine introduction. There was no secular trend in the incidence of radiologically-confirmed pneumonia over 8 years of study. After adjustment for secular trend and season, incidence rate ratios for admission with radiologically-confirmed pneumonia, clinically-defined pneumonia, and diarrhoea (control condition), associated temporally with PCV10 introduction and the catch-up campaign, were 0·52 (95% CI 0·32–0·86), 0·73 (0·54–0·97), and 0·63 (0·31–1·26), respectively. Immediately before PCV10 was introduced, the annual incidence of clinically-defined pneumonia was 1220 per 100 000; this value was reduced by 329 per 100 000 at the point of PCV10 introduction.

**Interpretation:**

Over 13 years, admissions to Kilifi County Hospital for clinically-defined pneumonia decreased sharply (by 27%) in association with the introduction of PCV10, as did the incidence of radiologically-confirmed pneumonia (by 48%). The burden of hospital admissions for childhood pneumonia in Kilifi, Kenya, has been reduced substantially by the introduction of PCV10.

**Funding:**

Gavi, The Vaccine Alliance and Wellcome Trust.

## Introduction

After the neonatal period, pneumonia is the greatest cause of death in children younger than 5 years^1^ and, before the introduction of pneumococcal conjugate vaccines (PCVs), the most common cause of fatal pneumonia was *Streptococcus pneumoniae* (pneumococcus).^2^ PCVs are highly efficacious against invasive pneumococcal disease caused by vaccine serotypes and their introduction in high-income countries has decreased transmission of vaccine serotypes and reduced invasive pneumococcal disease among vaccinated and unvaccinated populations. However, invasive pneumococcal disease represents only a small fraction of the burden of pneumococcal disease. For example, in a randomised controlled trial^3^ of 9-valent PCV (PCV9) in The Gambia, 15 cases of radiologically-confirmed pneumonia were prevented for every two cases of invasive pneumococcal disease.

**Research in context**

**Evidence before this study**

We searched PubMed for articles published between inception and Sept 1, 2018, using the MeSH keywords: “pneumonia, pneumococcal/prevention and control” AND “vaccines, conjugate” AND (“child” OR “infant”) AND (“South America” OR “central America” OR “Asia”, western” OR “Asia, southeastern” OR “Africa, south of the Sahara”), with no language restrictions. Our search showed that three randomised controlled trials of pneumococcal conjugate vaccines (PCVs) among children aged 0–2 years old in low-income and middle-income countries have examined radiologically-confirmed pneumonia as an endpoint. The efficacy of a 9-valent PCV was 37% (95% CI 25–48) in The Gambia, and 20% (2–35) in South Africa among HIV uninfected children. In the Philippines, the 95% CI for vaccine efficacy of an 11-valent PCV was −1% to 41% with a point estimate of 23%. Against clinically-defined severe pneumonia, PCV9 had an efficacy estimate of 17% (4–27) in South Africa (HIV uninfected) and 12% (−9 to 29) in The Gambia. PCV11 was not protective against severe pneumonia in the Philippines. These trials do not capture herd protection, which is a strong feature of the use of PCV in practice, or the effect on older children aged 2–4 years. The introduction of PCV7 in the USA caused a 39% reduction in admissions to hospital for pneumonia among children younger than 2 years; however, in low-income and middle-income countries, the magnitude of the effect is less clear. In The Gambia, the introduction of PCV7/PCV13 led to a 24–31% reduction in radiologically-confirmed pneumonia, but the effect on clinically-defined pneumonia was 5–15%, dependent on age. A retrospective case-review study of admission to hospital with pneumonia in Rwanda estimated that vaccine effectiveness of PCV7 was as high as 54%. A randomised controlled trial of 10-valent PCV, done in Argentina, Colombia and Panama, showed a vaccine efficacy against radiologically-confirmed pneumonia of 25·7%.

**Added value of this study**

This study, with its long time-span, consistency of methods, and rapid introduction of vaccination to children younger than 5 years with a catch-up campaign, provides robust evidence of the effect of PCV on pneumonia in tropical Africa. It is the first study to show the effect of PCV10 in a low-income setting. The introduction of PCV10 was associated with a reduction in admissions to hospital with clinically-defined pneumonia of 27% and with radiologically-confirmed pneumonia of 48%, among children aged 2–59 months. These effects are larger than those seen in randomised controlled trials, and are compatible with the development of herd protection. Our findings show that *Streptococcus pneumoniae* was responsible for at least 27% of all admissions to hospital with pneumonia in the pre-vaccine era and that the introduction of PCV10 has led to a substantial decline in childhood morbidity in Kenya and in the burden of hospital admissions.

**Implications of all the available evidence**

Pneumonia is the greatest cause of child death outside the neonatal period. It is also the most common manifestation of pneumococcal disease and, therefore, is the strongest driver of vaccine cost-effectiveness analyses. The evidence of a substantial effect of PCV10 against pneumonia will underpin policy making in African countries as they confront the challenge of sustaining PCV programmes independently from Gavi.

In low-income and middle-income countries, the efficacy of PCV against clinically-defined pneumonia is lower (0–17%) than the efficacy against invasive pneumococcal disease or radiologically-confirmed pneumonia.^3^, ^4^, ^5^ This finding suggests that clinically-defined pneumonia as an endpoint has poor specificity for pneumococcal pneumonia. WHO developed a set of interpretive criteria and procedures to standardise the reading of paediatric chest radiographs in pneumonia cases,^6^, ^7^ which defined an endpoint that has increased specificity for pneumococcal pneumonia and commensurately increased estimates of vaccine efficacy (20–37%).^3^, ^4^, ^8^, ^9^

Longitudinal studies of disease incidence, with an interrupted time-series analysis, are likely to capture the beneficial effects of herd protection due to reduced transmission of vaccine-serotype pneumococci and the effects of direct vaccine protection. These studies are also sensitive to serotype replacement disease if infection with non-vaccine serotypes leads to pneumonia. To date, there have been only two field studies of PCV effectiveness against pneumonia in low-income settings; one study^10^ had just 2 years of pre-vaccine surveillance, the other had none.^11^

In the USA, the effect of PCV7 on routine hospital admissions with all-cause pneumonia was estimated, using interrupted time-series analysis, to be a 39% reduction in children younger than 2 years, which is substantially greater than the efficacy estimates against clinically-defined pneumonia or radiologically-confirmed pneumonia in a randomised controlled trial.^12^, ^13^, ^14^ Unfortunately, in most low-income settings, the quality of routine administrative hospital data is insufficient for evaluation with this study design.

In an interrupted time-series analysis, after adjusting for seasonal and temporal trends in pneumonia hospitalisation, the residual change in incidence associated with vaccine introduction is as robust an estimate of vaccine impact as is possible in a non-randomised design.^15^, ^16^ Here, we aimed to use this method to capture the effect of PCV10 on clinically-defined and radiologically-confirmed pneumonia in a unique clinical and demographic surveillance platform in Kenya with real-time monitoring of vaccine coverage.^17^, ^18^ We introduced PCV10 with a catch-up campaign for children younger than 5 years to increase temporal specificity of the time-series effect.

## Methods

### Study design and participants

We studied residents (aged ≥2 months to <12 years) of the Kilifi Health and Demographic Surveillance System (KHDSS). KHDSS has monitored births, deaths, and migration events in a population of 280 000 through 4-monthly household visits since 2001.^18^

Kilifi County Hospital (Kilifi, Kenya) is centrally located within KHDSS and is the only paediatric inpatient facility in the study area. It has 55 paediatric beds. Since 2002, all admissions have been recorded using a standard electronic clinical record linked to the KHDSS population register.

We used WHO definitions of clinical pneumonia applicable at the start of the surveillance based on presentation with cough or difficulty breathing.^19^ Those with lower chest-wall indrawing but no danger signs had an admission diagnosis of severe pneumonia; those with at least one danger sign had very severe pneumonia. Danger signs were central cyanosis, inability to drink, convulsions, lethargy, prostration, unconsciousness, or head nodding.^19^ Since there were no specific definitions for children aged 5–11 years, we applied the same criteria as for younger children. Children with non-severe pneumonia are not normally admitted and were not included in the surveillance. Admission to hospital with diarrhoea was the control condition. Diarrhoea was defined as at least three loose stools in the past 24 hours.

From April, 2006 onwards, children with WHO-defined severe or very severe pneumonia were investigated, whenever possible, with a single frontal chest radiograph. Presentation with convulsions or lethargy alone, without other signs of pneumonia, was not considered by the local ethical review committee to be sufficient justification for investigation with a chest radiograph.

Written informed consent was obtained from the parents or guardians of all participants in the study. The study was approved by the KEMRI National Ethical Review Committee and Oxford Tropical Research Ethics Committee.

### Radiological reading and interpretation

Chest radiographs were taken with a Philips Cosmos-BS machine throughout the study period. A Philips Practix 360 portable machine became available in March, 2012. The radiology system was digitised in August, 2011; thereafter, a Philips PCR Eleva-S was used to process digital cassettes (10 × 12 inches; 1670 × 2010 pixels). Archived film images were digitised using a Vidar Pro Advantage digitiser. Images were encoded using Hipax software into DICOM format at 150 dpi and 12 bits. All images were cropped to de-identify patients and remove peripheral clues about the radiological method used, before being distributed in JPEG format in batches of 100 selected at random from pre-vaccine and post-vaccine introduction images.

Radiological interpretation followed the standard defined by WHO for the identification of primary endpoint pneumonia, which was used in the phase 3 trials of PCVs.^6^, ^7^ We categorised images by quality: adequate, suboptimal, or uninterpretable. Uninterpretable images were not assigned a diagnosis. Primary endpoint pneumonia was defined by the presence of consolidation or pleural effusion, or both.

Each image was read independently by two primary readers: a consultant radiologist and a trainee paediatrician in Kenya.^20^ All images with discordant interpretations, and 13% of those with agreement (as per our previous work^20^), were referred to three consultant radiologists in Oxford, UK, who arbitrated the readings. Concordant readings of the primary readers were considered final.

### Vaccine introduction and monitoring

In January, 2011, a 10-valent PCV (Synflorix; GlaxoSmithKline Biologicals, Rixensart, Belgium), consisting of capsular polysaccharides of serotypes 1, 4, 5, 6B, 7F, 9V, 14, 18C, 19F, and 23F conjugated to either non-typeable *Haemophilus influenzae* (NTHi) protein D, diphtheria, or tetanus toxoid, was introduced in Kenya in three doses at weeks 6, 10, and 14. There was a three-dose catch-up campaign for infants during 2011. In addition, in Kilifi County, children aged 12–59 months were offered two doses of vaccine via campaigns on Jan 31 to Feb 6, 2011, and March 21–27, 2011.

*Haemophilus influenzae* type b conjugate vaccine was introduced in Kenya in 2001. Rotavirus vaccine (Rotarix; GlaxoSmithKline Biologicals) was introduced, without a catch-up campaign, in July, 2014.

Vaccine surveillance was established in April, 2009, in 26 vaccine clinics serving KDHSS.^17^ Data clerks recorded all immunisations given against the identity of the child in the KHDSS population register at the point of vaccination. However, during the catch-up campaign, vaccinations were recorded against lists of KHDSS residents.

### Statistical analysis

The incidence of admission to hospital with clinically-defined pneumonia or diarrhoea was calculated for each month between May, 2002, and March, 2015. Children who had both pneumonia and diarrhoea were classified as having pneumonia alone. The monthly incidence of radiologically-confirmed pneumonia was calculated between April, 2006, and March, 2014. Mid-month population counts from the KHDSS were used to estimate child years at risk in each month.

We fitted linear regression models to log-transformed monthly rates of radiologically-confirmed and clinically-defined pneumonia to estimate the effect of PCV10. The models included a period effect (pre-PCV10 *vs* post-PCV10), monthly time trend, and seasonality, which was modelled using the month of the year. Differences in the time trends before and after vaccination were tested through the inclusion of an interaction term. We modelled the error as an autoregressive moving average process, using Aikake's information criterion and plots of the autocorrelation function of residuals to choose the order of the process.^21^ After observing the results of the analysis of the control condition, we further analysed the effect of PCV10 on clinically-defined pneumonia by adjusting for monthly incidence of diarrhoea admissions, instead of time in months. Because of the opposite pattern of seasonal incidence for diarrhoea and clinically-defined pneumonia (appendix), we deseasonalised the log-diarrhoea series by subtracting trend-adjusted estimates for the effect of each month. We smoothed the data using locally weighted scatterplot smoothing.

We excluded January to March, 2011, as a transition period during which PCV10 was introduced among children younger than 5 years. We also excluded admissions in December, 2012, and December, 2013, because of nurses' strikes.

When radiographs were not obtained, we used multiple imputations based on information on admission year, month of admission, sex, age, HIV status, malaria slide positivity, pneumonia severity, outcome of hospital admission (alive at discharge or died), referral, and day of the week when the patient was admitted. We created 20 imputed datasets, via chained equations,^22^, ^23^ and used Rubin's rules to combine estimates across the imputed datasets.

We calculated vaccine effectiveness as 1 – incidence rate ratio (IRR) using the IRRs estimated from the regression model for each disease classification. We estimated the absolute reduction in admission rates by multiplying the vaccine effectiveness estimates against the model predictions of incidence in the month immediately before the introduction of PCV10 in December, 2010. All statistical analyses were using STATA software (version 14).

### Role of the funding source

The funders of the study had no role in study design, data collection, data analysis, data interpretation, or writing of the report. The corresponding author had full access to all the data in the study and had final responsibility for the decision to submit for publication.

## Results

In May, 2002, there were 37 556 residents in KHDSS aged 2–59 months and 44 672 residents aged 60–143 months. By March, 2015, these figures were 45 601 and 62 502, respectively. By March 31, 2011, 4997 (61·1%) of 8181 children aged 2–11 months had received at least two doses of PCV10 and 23298 (62·3%) of 37416 children aged 12–59 months had received at least one dose of PCV10. Coverage increased throughout the rest of the study period (table 1).

**Table 1:** Demographics of population under observation within the Kilifi Health and Demographic Surveillance System

		2002*	**2003**	**2004**	**2005**	**2006**	**2007**	**2008**	**2009**	**2010**	**2011**	**2012**	**2013**	**2014**	**2015**†
**Population size (person-years)**															
2–11 months		4414	6789	6837	7103	7830	7385	7533	8047	7152	8058	7604	7401	7930	1891
12–23 months		5087	7834	8894	8657	8745	9565	9114	9649	9927	8854	9954	9269	9178	2435
24–59 months		15 686	23 805	24 731	25 485	25 738	26 137	26 568	27 042	28 200	28 463	28 263	28 684	27 835	6951
60–143 months		29 979	45 552	47 917	50 617	52 259	53 579	55 064	56 339	57 439	58 899	59 605	61 052	62 596	15 604
**Vaccinated with PCV (%)**‡															
2–11 months (≥2 doses)		..	..	..	..	..	..	..	..	..	79·9	76·4	81·4	87·7	84·2
12–23 months (≥1 dose)		..	..	..	..	..	..	..	..	..	76·0	84·4	85·6	89·3	91·6
24–59 months (≥1 dose)		..	..	..	..	..	..	..	..	..	62·7	66·9	74·6	82·8	86·5
60–143 months (≥1 dose)		..	..	..	..	..	..	..	..	..	7·2	15·7	24·1	32·4	42·8
**Admission to hospital**															
All causes															
	2–59 months	1131	2463	2315	1870	2006	1703	1578	1724	1455	869	1040	646	1049	257
	60–143 months	208	389	359	393	404	348	342	376	418	294	307	252	448	139
Severe pneumonia															
	2–59 months	167	420	521	443	535	382	321	334	330	154	213	97	145	49
	60–143 months	20	34	41	36	35	26	18	18	28	15	9	10	13	3
Very severe pneumonia															
	2–59 months	263	463	445	264	277	306	273	321	281	140	209	142	230	40
	60–143 months	25	45	36	28	41	30	23	28	43	14	23	30	43	8
Diarrhoea§															
	2–59 months	130	413	480	348	422	301	283	427	218	203	145	86	132	8
	60–143 months	8	17	19	19	16	16	11	33	26	7	15	5	12	2
Positive malaria slide															
	2–59 months	609	1,166	765	480	391	231	184	112	193	145	134	91	221	62
	60–143 months	109	188	106	94	106	54	38	36	83	84	84	83	155	53
Severe undernutrition¶															
	2–59 months	169	411	431	345	393	263	254	335	235	152	153	115	160	39
	60–143 months	34	48	61	58	59	40	39	59	56	23	57	26	50	17
**Months of pneumonia observation in the study**															
Clinically defined		8	12	12	12	12	12	12	12	12	9	11	11	12	3
Radiologically confirmed		0	0	0	0	9	12	12	12	12	9	11	11	3	0
**Number of pneumonia admissions with radiographs obtained**															
2–59 months		..	..	..	..	332	401	360	281	253	185	310	163	57	..
60–143 months		..	..	..	..	25	26	19	18	17	12	13	29	5	..
**Number of admissions with radiologically-confirmed pneumonia**															
2–59 months		..	..	..	..	76	94	70	68	65	28	46	49	21	..
60–143 months		..	..	..	..	11	6	6	6	6	4	5	8	3	..

Population estimates are mid-year populations. In 2002 and 2015, these are multiplied by the proportion of the year under observation. PCV=pneumococcal conjugate vaccine.

* 2002 includes only May to December.

† 2015 includes only January to March.

‡ Vaccine coverages are estimated in the last week of each year except 2015 (last week of March 2015).

§ Of 5229 patients with diarrhoea, 1427 also had clinically-defined pneumonia were classified only as pneumonia.

¶ Severe undernutrition was defined as a weight-for-age Z score of less than −3 on admission to hospital.

**Figure 1:**
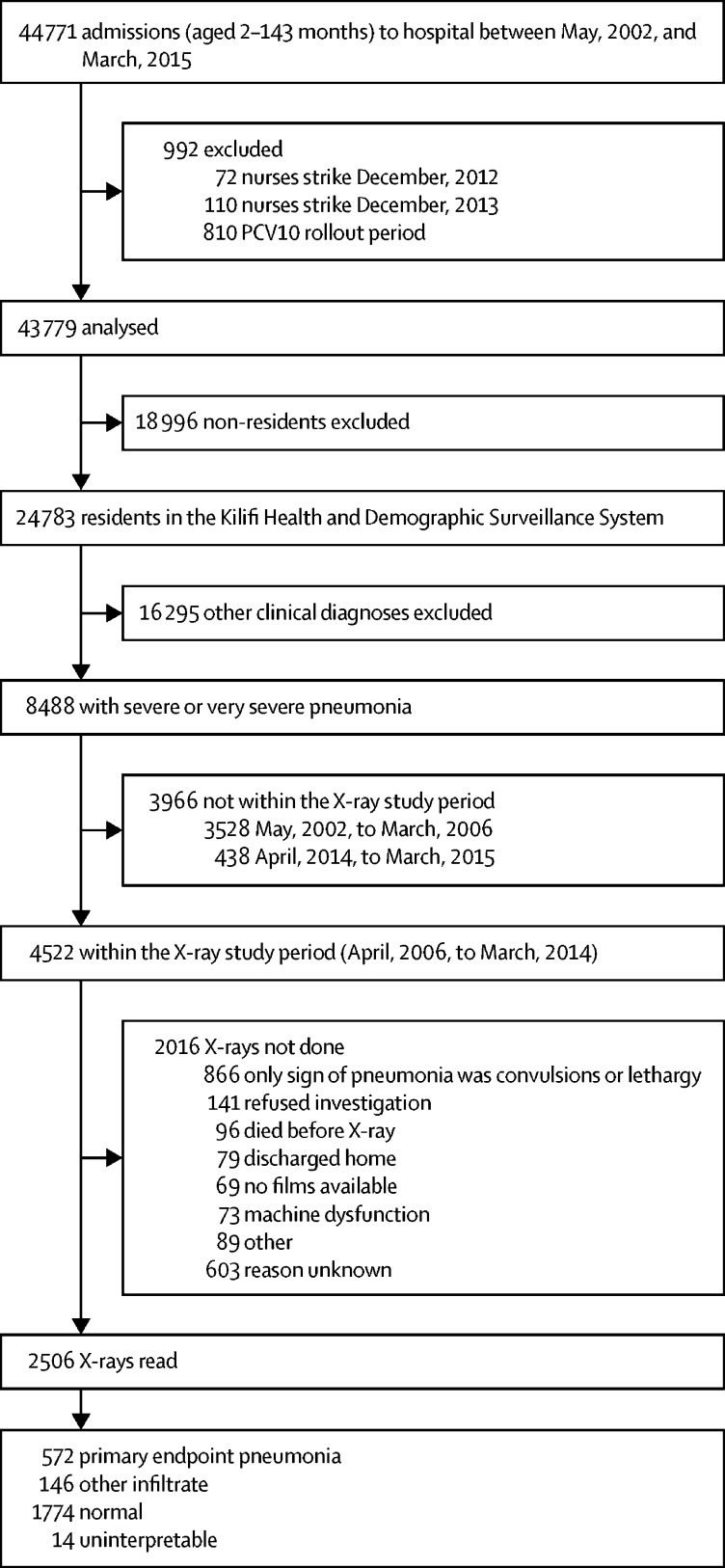
Flow chart showing the selection of patients for radiographs and the reasons for missing radiographs

Between May 1, 2002, and March 31, 2015, 44 771 children aged 2–143 months were admitted to Kilifi County Hospital (table 1). We excluded 810 admissions during the PCV10 introduction and catch-up campaign period (between January, 2011, and March, 2011), and 182 admissions during nurses' strikes. Of the remaining 43 779 admissions, 24 783 (57%) were residents of KHDSS (figure 1); of these, 8488 (34%) had severe or very severe clinically-defined pneumonia. Throughout the 13-year study period, the number of admissions to hospital among KHDSS residents aged 2–59 months fell progressively, particularly those with severe undernutrition (defined as weight for age Z score less than −3) and a positive malaria slide (table 1). The prevalence of HIV infection among mothers attending the antenatal clinic in Kilifi County Hospital was 4·1% in 2005–07, 4·2% in 2008–10, and 2·7% in 2011–15.

Among pneumonia patients, 4522 (53%) were admitted during the radiological study period (April, 2006, to March, 2014) and at least one chest radiograph was obtained from 2506 (55%). Radiographs were obtained from 51% (1732 of 3373) of patients in the pre-vaccine period and 67% (774 of 1149) in the post-vaccine period. During the pre-vaccination period (ie, before Jan 1, 2011), primary endpoint pneumonia was identified in 21% (185 of 867) of readable radiograph images among children aged 2–11 months, 26% (109 of 423) among children aged 12–23 months, 24% (79 of 327) among children aged 24–59 months, and 34% (35 of 104) among children aged 60–143 months.

**Figure 2:**
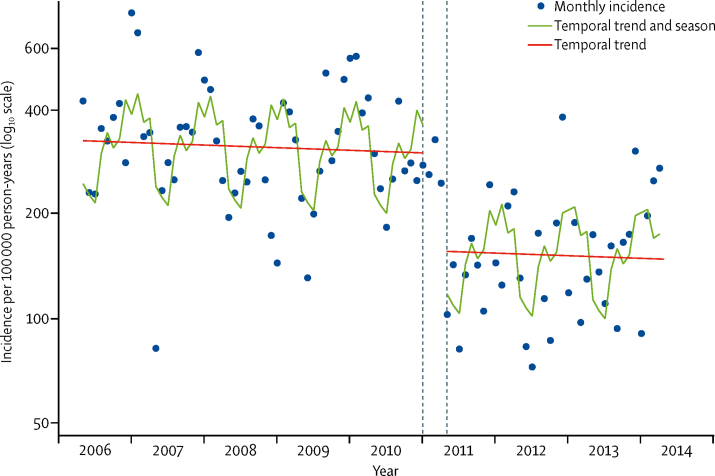
Monthly incidence of admission to Kilifi County Hospital among children aged 2–59 months with WHO-defined radiologically-confirmed pneumonia and modelled predictions The dashed lines show the transition period during which PCV10 was introduced among children younger than 5 years. The model excluded datapoints for December, 2012, and December, 2013, to account for two nurses' strikes at Kilifi County Hospital.

Before imputation and modelling, the crude incidence rates for radiologically-confirmed pneumonia among children aged 2–59 months were 180·9 (95% CI 163·0–200·2) per 100 000 person-years for the period before PCV10 was introduced and 110·7 (93·3–130·3) for the period after PCV10 introduction; the crude IRR was 0·61 (0·50–0·74). In the interrupted time-series model, which adjusted for season and time (figure 2, table 2), the IRR for radiologically-confirmed pneumonia associated with PCV10 introduction among children aged 2–59 months was 0·52 (0·32–0·86). The effect was greatest among those aged 12–59 months. There was no effect among children aged 60–143 months (table 2). Radiologically-confirmed pneumonia varied substantially by season, with a peak in November to January and a trough in April to June (appendix). After accounting for season and the vaccine effect, the underlying incidence of radiologically-confirmed pneumonia among children 2–59 months was stable over time (IRR per month 0·999, 0·990–1·007; appendix).

**Table 2:** IRRs for the effects of PCV10 introduction on admission to hospital with radiologically-confirmed pneumonia among children aged 2–143 months, by age subgroup and clinical severity

		**IRR for PCV10 introduction**	**95% CI**	**p value**
**Age**				
2–59 months (primary analysis)		0·52	0·32–0·86	0·011
	2–11 months	0·73	0·39–1·36	0·324
	12–23 months	0·54	0·27–1·10	0·091
	24–59 months	0·50	0·24–1·01	0·052
60–143 months		0·89	0·47–1·69	0·724
**Clinical severity (2–59 months only)**				
Severe pneumonia		0·56	0·30–1·06	0·077
Very severe pneumonia		0·52	0·28–0·96	0·038

Interrupted time series analysis adjusted for season (month of year) and study time (months). IRRs=incidence rate ratios.

**Figure 3:**
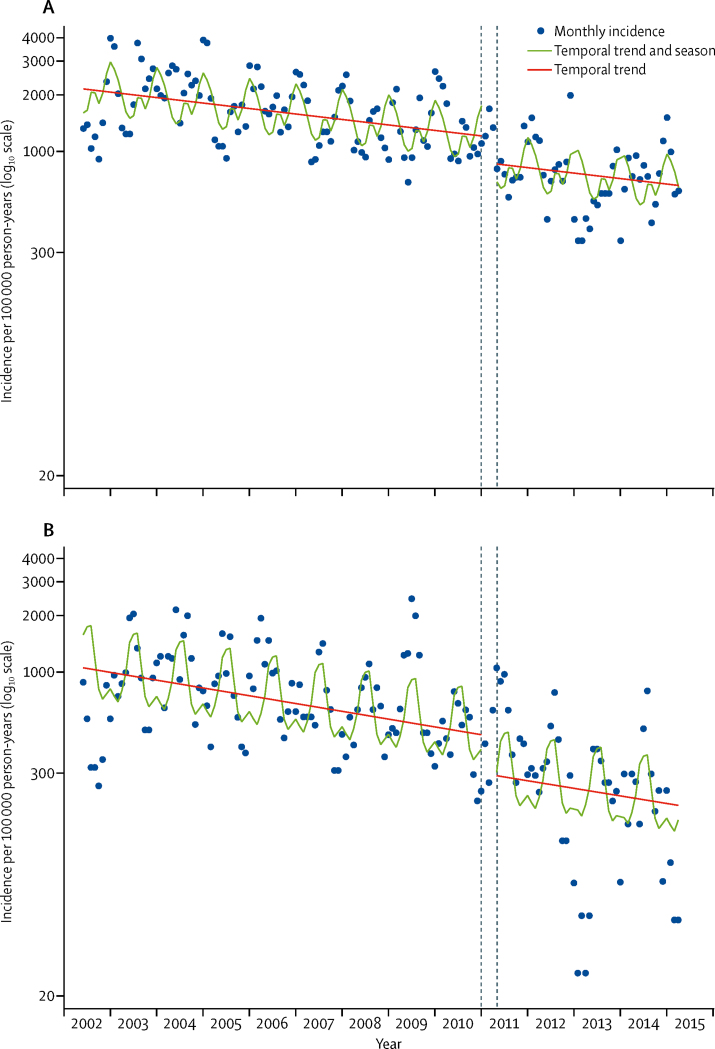
Monthly incidence of admission to Kilifi County Hospital among children aged 2–59 months with (A) clinically-defined pneumonia and (B) diarrhoea Clinically-defined pneumonia includes severe or very severe pneumonia, according to WHO definitions.^19^ The dashed lines show the transition period during which PCV10 was introduced among children younger than 5 years. The model excluded datapoints for December, 2012, and December, 2013, to account for two nurses' strikes at Kilifi County Hospital.

The annual incidence of admission with clinically-defined pneumonia in 2002–03 was 2170 per 100 000 in children aged 2–59 months; incidence of admission to hospital significantly reduced across the study period by 0·5% per month (figure 3). Pneumonia admissions also had marked seasonal variation, which closely followed that of radiologically-confirmed pneumonia (appendix). After adjusting for these factors, the IRR for admissions with severe or very severe pneumonia associated with introduction of the PCV10 programme was 0·73 (95% CI 0·54–0·97; table 3). Vaccine impact was greater for severe pneumonia than for very severe pneumonia (table 3). There was no evidence of benefit to children aged 60–143 months.

**Table 3:** IRRs for the effects of PCV10 introduction on admission to hospital with WHO-defined severe or very severe pneumonia among children aged 2–143 months

		**Severe pneumonia**			**Very severe pneumonia**			**All pneumonia**		
		IRR	95% CI	p value	IRR	95% CI	p value	IRR	95% CI	p value
**Adjusted for time**										
2–59 months		0·60	0·40–0·91	0·017	0·87	0·56–1·34	0·519	0·73	0·54–0·97	0·033
	2–11 months	0·66	0·41–1·07	0·090	0·73	0·48–1·11	0·143	0·70	0·50–1·00	0·048
	12–23 months	0·61	0·39–0·94	0·027	1·28	0·83–1·96	0·264	0·84	0·61–1·15	0·283
	24–59 months	0·59	0·37–0·92	0·020	0·78	0·40–1·54	0·479	0·71	0·43–1·19	0·192
60–143 months		0·93	0·62–1·39	0·721	0·96	0·61–1·50	0·857	0·95	0·56–1·59	0·832
**Adjusted for diarrhoea admissions**										
2–59 months		0·95	0·60–1·51	0·818	0·74	0·36–1·51	0·405	0·83	0·54–1·28	0·399
	2–11 months	0·91	0·55–1·48	0·697	0·72	0·43–1·21	0·219	0·86	0·60–1·24	0·428
	12–23 months	1·07	0·64–1·77	0·804	1·32	0·69–2·53	0·408	1·10	0·71–1·71	0·681
	24–59 months	0·76	0·46–1·27	0·299	0·68	0·32–1·43	0·308	0·76	0·43–1·33	0·334
60–143 months		1·14	0·41–3·18	0·795	0·51	0·25–1·06	0·072	0·61	0·18–2·05	0·427

Interrupted time-series analysis adjusted for season (month of year) and temporal trends (time in months or natural log-transformed monthly incidence rates of admission with diarrhoea). We deseasonalised the log-diarrhoea series by subtracting trend-adjusted estimates for the effect of each month, and smoothed the series using locally weighted scatterplot smoothing. IRRs=incidence rate ratios.

There was no interaction between study time and vaccine era in the analysis of the incidence of clinically-defined pneumonia, nor of radiologically-confirmed pneumonia among children aged 2–59 months. This finding indicates that there was no further development of indirect protection after the catch-up campaign.

After adjusting for seasonal variation and secular trends, the modelled incidence rate of clinically-defined pneumonia in December, 2010, was 1220 per 100 000 person-years among children aged 2–59 months; for radiologically-confirmed pneumonia, this value was 301 per 100 000 person-years. By multiplying these incidence rates against the vaccine effectiveness estimates for clinically-defined and radiologically-confirmed pneumonia, the reduction in disease incidence attributable to vaccine introduction was 329 and 144 cases per 100 000 person-years, respectively.

The control condition—incidence of admission with diarrhoea among children aged 2–59 months—was not associated with PCV10 introduction (IRR 0·63, 95% CI 0·31–1·26; figure 3). However, in age-stratified analyses, there was an association between PCV10 introduction and incidence of diarrhoeal admissions in those aged 12–23 months (0·63, 0·41–0·99), 24–59 months (0·62, 0·39–0·99), and 60–143 months (0·66, 0·46–0·95; appendix). The IRR among infants (0·79) was less extreme than the IRRs seen in older age groups (0·62–0·66). Truncating analysis time at the point when rotavirus vaccination was introduced did not alter these findings.

We explored whether the observed effect of PCV10 on diarrhoea in some age groups suggested residual confounding in patterns of hospital presentation. To select a suitable control condition, we examined the correlation between annual counts of admissions with clinically-defined pneumonia in the pre-vaccine period against annual counts of admissions with other discharge diagnoses. The greatest correlations were with unclassified discharges and with gastroenteritis, although unclassified discharges were relatively uncommon. The correlation with admission diagnosis diarrhoea was greater yet (appendix). After adjusting for log-transformed monthly rates of diarrhoea admissions, instead of time in months, the IRR for clinically-defined pneumonia associated with PCV10 in children aged 2–59 months was 0·83 (95% CI 0·54–1·28, table 3).

## Discussion

This interrupted time-series analysis of the rates of hospital admission from a rolling cohort of about 43 000 children aged 2–59 months in Kilifi, Kenya, suggests that the introduction of PCV10, with a simultaneous catch-up campaign for children younger than 5 years, was associated with a reduction in childhood admissions to hospital with clinically-defined pneumonia (by 27%) and radiologically-confirmed pneumonia (by 48%). The vaccine reduced the incidence of admission to hospital with clinically-defined pneumonia (by 329 per 100 000 person-years) and radiologically-confirmed pneumonia (by 144 per 100 000 person-years). There was no effect among children aged 5 years or older.

The observed effect in Kilifi was considerably greater than the vaccine efficacy estimates from individually randomised controlled trials of PCVs. Against severe clinically-defined pneumonia, the vaccine efficacy of a 9-valent PCV was 12% (95% CI −9 to 29) in The Gambia^3^ and 17% (7 to 26) in South Africa.^5^ Against radiologically-confirmed pneumonia, the vaccine efficacies were 37% in The Gambia^3^ and 20% in South Africa.^8^ In Bohol, the Philippines, the point estimate for vaccine efficacy of an 11-valent PCV against radiologically-confirmed pneumonia was 22·9% (−1·1 to 41·2); there was no protection against clinically-defined pneumonia.^4^ A randomised controlled trial^9^ of PCV10, done in Argentina, Panama, and Colombia, estimated vaccine efficacy against radiologically-confirmed pneumonia at 22·4%. These vaccine efficacy estimates, derived from individually randomised trials, measure only the direct protective effect of the vaccine, whereas the impact estimates in the present study combine direct and indirect effects. In the USA, the indirect effect of PCV7 against pneumonia was substantial;^24^ it is not surprising, therefore, that the impact estimates in a real-world implementation in Africa are considerably greater than the efficacy estimates from trials.

There is relatively little information on the effect of PCV elsewhere in tropical Africa. In a retrospective analysis^25^ (2002–12) of clinically-defined pneumonia among admission case-records from five district hospitals in Rwanda, the impact of PCV7, introduced in 2009, was similar to that observed in Kilifi (vaccine effectiveness 54%, 95% CI 42–63). However, a study of the effect of PCV in children younger than 5 years in The Gambia, comparing rates in the post-PCV13 era (2014–15) with those in the pre-PCV7 era (2008–10), found a 5–15% reduction in hospitalised clinically-defined pneumonia, depending on age. For children admitted to hospital with radiologically-confirmed pneumonia, the reductions were 24–31%,^10^ which were lower than the 37% estimate derived from a randomised controlled trial^3^ of PCV9 in the same setting.

In Kilifi, across the 13-year study period, admission incidence rates were consistently reduced, particularly for malaria, malnutrition, and clinically-defined pneumonia. Over the same period, mortality ratios in infants and children younger than 5 years decreased substantially, suggesting that changing admission rates reflect a genuine improvement in health, rather than a change in health-seeking behaviour. The effect of general health trends on the incidence of clinically-defined pneumonia in the pre-vaccine period was greater than the effect of PCV10 introduction in 2011. Among children aged 2–59 months, the annual incidence of clinically-defined pneumonia per 100 000 children declined from 2170 in 2002–03 to 1220 in December, 2010; however, with the introduction of PCV10, this incidence declined further by 329. In other settings across Africa, which have a higher baseline incidence than in Kenya, the absolute benefits of PCV10 are probably considerably greater. However, the full magnitude of the effect might take longer in the absence of a catch-up campaign.

The observed effect of PCV10 against radiologically-confirmed pneumonia is substantially greater than that against clinically-defined pneumonia, suggesting that vaccine serotype pneumococci account for proportionately more cases of radiologically-confirmed pneumonia than of clinically-defined pneumonia. The WHO radiological standard was developed to generate an endpoint that was specific for bacterial pneumonia and, in the presence of a vaccine programme for *H influenzae* type b, it is relatively specific for pneumococcal pneumonia.^3^, ^6^ However, as well as differences in effect, we saw marked differences in temporal trends; radiologically-confirmed pneumonia was stable, whereas clinically-defined pneumonia declined sharply with time. Clinical presentations of pneumonia and malaria are difficult to distinguish^26^ and the prevalence of malaria has declined sharply from 1999 to 2007,^27^ suggesting that some of the temporal trends in clinically-defined pneumonia admissions might be attributable to changes in malaria incidence.

We estimated the absolute reduction in admissions with radiologically-confirmed pneumonia attributable to PCV10 introduction as 144 cases per 100 000 person-years. Separately,^20^ we have evaluated the performance of the study's chest-radiograph readers in Kenya by comparing their readings of 1179 images against the consensus interpretation of three experienced consultant radiologists in Oxford, UK. Although the specificity of the local readers for radiologically-confirmed pneumonia was high (0·95–0·96), the sensitivity was relatively low (0·69–0·73).^20^ This disparity is unlikely to affect the relative estimates of the effect of PCV10, expressed as IRRs, but it does suggest that we underestimated the absolute reduction in incidence of radiologically-confirmed pneumonia attributable to the vaccine programme by about 30%.

Following the example of previous studies, we selected diarrhoea as a control condition because it was common and should be unaffected by PCV10.^12^ However, we observed an unexpected decrease in diarrhoea admissions among children 2–59 months associated with the timing of PCV10 introduction (IRR 0·63, 95% CI 0·31–1·26). We examined whether there was residual confounding in the temporal pattern of hospital presentations by adjusting for diarrhoea admissions, instead of time in years. The point estimates for these incidence rate ratios are consistent with the hypothesis that the vaccine is protective, but the estimates lose statistical significance.

The decline in diarrhoeal admissions after PCV10 introduction is unlikely to be due to a biological effect of PCV10. However, it might be a marker of another intervention in this area that is targeting diarrhoea at the same time as PCV10 introduction. The effect was significant in all age groups beyond infancy and was especially marked in those aged 5–11 years, who were too old to be vaccinated with PCV10 (appendix). In 2007, a campaign of community-led total sanitation was introduced into Kilifi County to eliminate open defecation by encouraging behaviour change and building toilets. By 2012, 25% of the villages in Kilifi County had this programme. It is possible that the campaign had some effect on the incidence of diarrhoea at the same time as PCV10 introduction. If so, the use of diarrhoea as an adjustment variable for temporal trends in hospital presentation would underestimate the true effect of PCV10 against clinically-defined pneumonia. Despite the ambiguity of the diarrhoea results, PCV10 had a large and significant effect on radiologically-confirmed pneumonia in children younger than 5 years and the temporally-adjusted impact estimates for severe (IRR 0·60) and very severe clinically-defined pneumonia (IRR 0·87) are consistent with this finding.

The reduction, by 27%, in admissions with clinically-defined pneumonia implies that at least 27% of these admissions were attributable to pneumococcal infections of vaccine serotypes.^28^ As we inferred for radiologically-confirmed pneumonia, the greater effect of PCV10 on severe versus very severe clinically-defined pneumonia probably reflects the greater role of pneumococcus among cases of severe pneumonia compared with very severe pneumonia. Severe pneumonia was defined by lower chest wall indrawing, which is a marker of poor lung compliance during respiratory infection. Very severe pneumonia was diagnosed by danger signs used in the Integrated Management of Childhood Illness to define very severe disease.^19^ Children with danger signs, which included central cyanosis, inability to drink, convulsions, lethargy, prostration, or unconsciousness, probably include a reasonable proportion of admissions who have febrile convulsions, malaria, sepsis, meningitis, and encephalitis. Few of these children would have had an aetiology preventable by PCV10 and this observation might have diluted the estimate of vaccine effect against very severe clinically-defined pneumonia.

Several properties of the present study suggest that the associations observed between vaccine introduction and disease incidence were causal: the duration of surveillance was long and the surveillance methods were consistent; the vaccine programme was introduced with a rapid catch-up campaign and nearly two-thirds of the target population were given an immunising schedule at the same time point; the analyses accounted for long-term trends in disease incidence and seasonal variation; and the magnitude of the effects was large and therefore difficult to attribute to incidental unobserved improvements in the environment. In addition, these changes in the incidence of pneumonia occurred simultaneously with a 64% reduction in the prevalence of carriage of the serotypes included in the vaccine^29^ and with a 68% decline in the incidence of cases of invasive pneumococcal disease.^30^

Taken together, these factors suggest that the introduction of PCV10 in Kilifi, Kenya, has reduced the incidence of admissions to hospital with clinically-defined pneumonia by 27%, and of radiologically-confirmed pneumonia by 48%. Given that pneumonia, rather than invasive pneumococcal disease, causes the greatest burden of pneumococcal disease, these findings suggest that there is a considerable improvement in child health associated with the implementation of a PCV10 programme.
