# Supplementary material for: Effect of 10-valent pneumococcal conjugate vaccine on the incidence of radiologically-confirmed pneumonia and clinically-defined pneumonia in Kenyan children: an interrupted time-series analysis
Source: Lancet Glob Health. 2019 Feb 14;7(3):e337–46. doi: 10.1016/S2214-109X(18)30491-1 (PMC6379823; doi:10.1016/S2214-109X(18)30491-1)
Supplement: Supplementary appendix [file mmc1.pdf]

THE LANCET
Global Health

Supplementary appendix

This appendix formed part of the original submission and has been peer reviewed. We post it as supplied by the authors.

Supplement to: Silaba M, Ooko M, Bottomley C, et al. Effect of 10-valent pneumococcal conjugate vaccine on the incidence of radiologically-confirmed pneumonia and clinically-defined pneumonia in Kenyan children: an interrupted time-series analysis. *Lancet Glob Health* 2019; **7:** e337–46.

Supplementary Tables

The effect of 10-valent Pneumococcal Conjugate Vaccine on the incidence of radiologically-confirmed pneumonia and clinically-defined pneumonia in Kenyan children: an interrupted time-series analysis.

Micah Silaba, Michael Ooko, Christian Bottomley, Joyce Sande, Rachel Benamore, Kate Park, James Ignas, Kathryn Maitland, Neema Mturi, Anne Makumi, Mark Otiende, Stanley Kagwanja, Sylvester Safari, Victor Ochola, Tahreni Bwanaali, Evasius Bauni, Fergus Gleeson, Maria Deloria Knoll, Ifedayo Adetifa, Kevin Marsh, Thomas N Williams, Tatu Kamau, Shahnaaz Sharif, Orin S Levine, Laura L Hammitt, J Anthony G Scott

<u>**Contents**</u>

Page 2: Suppl. Table 1

Page 3: Suppl. Table 1

Page 4: Suppl. Table 1

Suppl. Table 1. Incidence rate ratios for season, time and PCV10 introduction against hospitalisation with radiologically-confirmed pneumonia, with severe or very severe clinically-defined pneumonia, and with diarrhoea among children aged 2-59 months.

	Radiologically-confirmed pneumonia		Severe or very severe clinically-defined pneumonia		Diarrhoea	
**Variable**	**Incidence Rate Ratio**	**95% CI**	**Incidence Rate Ratio**	**95% CI**	**Incidence Rate Ratio**	**95% CI**
PCV10 introduction	0·52	0·32, 0·86	0·73	0·54, 0·97	0·63	0·31, 1·26
Time (in months)	0·999	0·990, 1·007	0·995	0·991, 0.998	0·992	0·985, 0.999
Seasonal month						
January	Ref		Ref		Ref	
February	0·83	0·47, 1·46	0·88	0·74, 1·05	0·94	0·71, 1·24
March	0·85	0·47, 1·56	0·73	0·58, 0·93	1·08	0·80, 1·46
April	0·54	0·32, 0·92	0·61	0·48, 0·77	1·34	0·88, 2·06
May	0·51	0·27, 0·94	0·57	0·44, 0·73	1·97	1·29, 2·99
June	0·48	0·25, 0·93	0·59	0·46, 0·76	2·18	1·46, 3·26
July	0·67	0·33, 1·33	0·73	0·58, 0·93	2·24	1·48, 3·38
August	0·77	0·42, 1·39	0·73	0·57, 0·94	1·52	0·92, 2·49
September	0·69	0·37, 1·31	0·65	0·50, 0·84	1·06	0·67, 1·66
October	0·74	0·40, 1·37	0·74	0·55, 0·99	0·94	0·62, 1·42
November	0·96	0·57,1·62	0·94	0·75, 1·18	1·01	0·73, 1·40
December	0·87	0·50,1·50	1·09	0·91, 1·29	1·08	0·78, 1·51

CI, confidence interval

Suppl. Table 2. Correlation between annual admission counts of clinically-defined pneumonia and discharge diagnoses of other diseases in the pre-vaccine era (2002-2010)

Diagnosis*	Number	Correlation	95% CI	P-value
At discharge				
Unclassified	274	0·85	0·42, 0·97	0·004
Gastroenteritis	2,146	0·81	0·32, 0·96	0·008
Encephalopathy	66	0·80	0·28, 0·96	0·010
Trauma	177	0·74	0·15, 0·94	0·023
Malnutrition	767	0·67	0·02, 0·92	0·046
Febrile convulsions	521	0·56	-0·16, 0·89	0·11
Meningitis	67	0·52	-0·22, 0·88	0·15
Malaria	2,308	0·48	-0·27, 0·87	0·19
Sepsis	89	0·45	-0·31, 0·86	0·23
All other conditions	632	0·40	-0·36, 0·84	0·28
Burns	369	0·35	-0·41, 0·82	0·36
Anaemia	399	0·32	-0·44, 0·81	0·41
Cellulitis	280	0·22	-0·52, 0·77	0·57
Kerosene poisoning	97	0·20	-0·53, 0·76	0·60
Upper Respiratory Infection	322	0·16	-0·56, 0·75	0·68
Epilepsy	121	0·08	-0·62, 0·71	0·84
Sickle cell disease	243	0·02	-0·65, 0·68	0·95
Elective surgery	132	-0·11	-0·72, 0·60	0·79
On admission				
Diarrhoea	3,010	0·89	0·57, 0·98	0·001

CI confidence interval

* Between 2002-2010 there were 28,000 paediatric admissions in the age group 2-59 months; 16,245 (58%) were residents of the KHDSS. Of these 6,346 had a clinical diagnosis of severe or very severe pneumonia. Of the remaining 9,899 children, 643 had a primary discharge diagnosis of lower respiratory tract infection (LRTI) and 272 had a secondary discharge diagnosis of LRTI; the discharge diagnoses of 70 children were missing. All of these were excluded from the analysis leaving 8,914 children with non-pneumonia diagnoses. As gastroenteritis was the classified category with the highest correlation, we also examined the correlation between admission with diarrhoea (but without severe or very severe pneumonia) and admission with pneumonia which is given in the last row of the table.

Suppl. Table 3. Incidence rate ratios for the effects of PCV10 introduction against hospitalisation with WHO-defined severe or very severe pneumonia among children aged 2-143 months, in sub-groups of age and clinical severity, and with diarrhoea and all other admissions.

End-point and sub-groups	PCV10 Incidence Rate Ratio	95% CI	P value
Severe pneumonia			
2-59 months	0·60	0·40, 0·91	0·017
Very severe pneumonia			
2-59 months	0·87	0·56, 1·34	0·519
Severe or very severe pneumonia			
2-59 months	0·73	0·54, 0·97	0·033
2-11 months	0·70	0·50, 1·00	0·048
12-23 months	0·84	0·61, 1·15	0·283
24-59 months	0·71	0·43, 1·19	0·192
60-143 months	0·95	0·56, 1·59	0·832
Diarrhoea			
2-59 months	0·63	0·31, 1·26	0·190
2-11 months	0·79	0·42, 1·48	0·459
12-23 months	0·63	0·41, 0·99	0·044
24-59 months	0·62	0·39, 0·99	0·046
60-143 months	0·66	0·46, 0·95	0·027
All other admissions			
2-59 months	0·89	0·57, 1·39	0·614

CI, confidence interval

The analyses of severe and very severe pneumonia have been presented previously in Table 3. They are reproduced here to compare with the incidence rate ratios in the control condition (diarrhoea).
